# Prevalence of the Recently Proposed Clinical Obesity Among Older Adults From the Health and Retirement Study

**DOI:** 10.1093/geroni/igaf122.1324

**Published:** 2025-12-31

**Authors:** Weiyang Ding, Ngai Kwan, Laurie Milliken

**Affiliations:** University of Massachusetts Boston, Boston, Massachusetts, United States; Institute for Community Inclusion, Boston, Massachusetts, United States; University of Massachusetts Boston, Boston, Massachusetts, United States

## Abstract

Obesity is traditionally defined as a body mass index (BMI) ≥30 kg/m² (CurDef), but this may overlook health risks, especially in older adults. The Lancet Diabetes and Endocrinology Commission on Obesity recently proposed a new definition (NewDef) that classifies individuals with BMI ≥30 kg/m² as “clinical obese” only if they also have high adiposity (measured by waist circumference, WC) and an obesity-related chronic condition. This study compared the prevalence of obesity using NewDef versus CurDef in a nationally representative sample of older adults (≥50 years) from the Health and Retirement Study (HRS) (waves 13 and 14; n = 13,852; 58% female). The prevalence of obesity was 45.9% (95% CI: 45.1–46.7) using CurDef and 40.4% (95% CI: 39.6–41.3) using NewDef, reducing the number of obese individuals by 754. Among those reclassified as non-obese, 26.6% (18.4% female) had BMI ≥30 and low WC, 19.6% (18.9% female) had BMI ≥30, low WC, and ≥1 chronic condition, and 73.3% (53% female) had high BMI and WC but no chronic condition. The NewDef lowers the prevalence of obesity and may better identify older adults at higher health risk by focusing on meaningful health consequences rather than BMI alone. This refined approach could improve clinical care and resource allocation for older adults. However, the lack of detailed information on whether chronic conditions are obesity-related limits the accuracy of the NewDef, highlighting the need for improved health condition documentation in older adult populations.

